# Letter to the editor: Critical need for robust surveillance in response to DENV-2 and SARS-CoV-2 cross-reactivity

**DOI:** 10.2807/1560-7917.ES.2024.29.19.2400236

**Published:** 2024-05-09

**Authors:** Chia Siang Kow, Dinesh Sangarran Ramachandram, Syed Shahzad Hasan, Kaeshaelya Thiruchelvam

**Affiliations:** 1 School of Pharmacy, International Medical University, Kuala Lumpur, Malaysia; 2 School of Pharmacy, Monash University Malaysia, Bandar Sunway, Selangor, Malaysia; 3 School of Applied Sciences, University of Huddersfield, Huddersfield, United Kingdom

**Keywords:** COVID-19, dengue virus, SARS-CoV-2

**To the editor**: The study by Klitting et al. [1], which investigates the phylogenetic analysis and geographic distribution of dengue virus serotype 2 (DENV-2) across various French territories, represents an advancement in our understanding of DENV epidemiology. By unravelling the evolutionary dynamics and transmission pathways of DENV-2, particularly across the French Caribbean Islands, French Guiana, Réunion, and mainland France, this research underscores the urgent need not only to strengthen comprehensive surveillance, particularly in and across endemic countries as well as non-endemic areas at risk for DENV introduction, but also to heighten public health vigilance.

Of significant concern is the study's revelation of the circulation of a specific DENV-2 lineage in French Guiana and mainland France, initially detected in the French Caribbean Islands. This lineage has been associated with widespread outbreaks and indigenous transmission, highlighting intricate regional transmission dynamics. Furthermore, the genetic relatedness of DENV-2 strains in autochthonous cases in mainland France to those from the Caribbean underscores the potential for inter-territorial spread facilitated by global travel.

Given these findings, we wish to highlight that the recent discoveries of strong binding of severe acute respiratory syndrome coronavirus 2 (SARS-CoV-2) antibodies to the DENV-2 envelope (E)-protein have emerged as a pressing issue [2]. This cross-reactivity may potentially lead to more DENV replication, worsening the course of dengue, a phenomenon recognised as antibody-dependent enhancement (ADE) [3]. ADE occurs when non-neutralising or sub-neutralising antibodies from a previous infection by – or vaccination against – a virus, bind to a different virus, such as DENV, and enhance viral entry into target cells, leading to increased viral replication and potentially more severe disease outcomes [4]. With the widespread prevalence of SARS-CoV-2 globally, the implications of the interaction between SARS-CoV-2 antibodies and DENV-2 are particularly concerning. Individuals who have been previously infected with or vaccinated against SARS-CoV-2 may have circulating antibodies that could potentially enhance the effects of DENV-2 infection if they are exposed to the virus. This may pose a risk, especially in areas where DENV is endemic, as it could lead to higher rates of severe dengue illness and complications among individuals with prior exposure to SARS-CoV-2.

The findings of these studies accentuate the need for intensified genomic and serological surveillance in areas affected by both DENV and SARS-CoV-2. Comprehending whether ADE can occur in relation to infection with these viruses is pivotal, as ADE may worsen the course of disease, influence vaccine effectiveness, and pose challenges to existing diagnostic methods. Furthermore, these insights should lead health authorities in endemic areas to reassess and adapt their public health strategies, considering the potential shift in disease dynamics. These strategies not only should include enhanced surveillance to track viral genotypes and mutations, but also efforts to better understand the immunological interactions arising from DENV and SARS-CoV-2 infections, particularly the role of prior SARS-CoV-2 infections and/or COVID-19 vaccination status on host response to DENV-2. This integrated approach will be essential for effectively managing the complex interplay between these two considerable viral pathogens.

In summary, the research on the circulation of the DENV-2 lineage across various French territories, coupled with the discovery of antibody cross-reactivity between SARS-CoV-2 and DENV-2, emphasises the important role of comprehensive viral surveillance. Such initiatives are crucial for addressing the intricate challenges posed by the co-circulation of these viruses and for implementing targeted public health interventions to effectively manage and prevent the spread of these infectious diseases within and across regions.
